# Structure and Doping Optimization of IDT-Based Copolymers for Thermoelectrics

**DOI:** 10.3390/polym12071463

**Published:** 2020-06-30

**Authors:** Tongchao Liu, Dexun Xie, Jinjia Xu, Chengjun Pan

**Affiliations:** 1 School of Chemistry, Sun Yat-sen University, Guangzhou 510275, China; liu21645@163.com; 2 Shenzhen Key Laboratory of Polymer Science and Technology, College of Materials Science and Engineering, Shenzhen University, Shenzhen 518060, China; pancj@szu.edu.cn; 3 Weldon School of Biomedical Engineering, Birck Nanotechnology Center, Center for Implantable Devices, Purdue University, West Lafayette, IN 47907, USA; xu.jinjia515@hotmail.com

**Keywords:** organic thermoelectric materials, conjugated polymer, indacenodithiophene (IDT), backbone engineering

## Abstract

π-conjugated backbones play a fundamental role in determining the thermoelectric (TE) properties of organic semiconductors. Understanding the relationship between the structure–property–function can help us screen valuable materials. In this study, we designed and synthesized a series of conjugated copolymers (**P1**, **P2**, and **P3**) based on an indacenodithiophene (IDT) building block. A copolymer (**P3**) with an alternating donor–acceptor (D-A) structure exhibits a narrower band gap and higher carrier mobility, which may be due to the D-A structure that helps reduce the charge carrier transport obstacles. In the end, its power factor reaches 4.91 μW m^−1^ K^−2^ at room temperature after doping, which is superior to those of non-D-A IDT-based copolymers (**P1** and **P2**). These results indicate that moderate adjustment of the polymer backbone is an effective way to improve the TE properties of copolymers.

## 1. Introduction

Thermoelectric (TE) materials, which can directly convert thermal energy into electrical energy, provide an environmentally friendly method for energy utilization [1,2,3,4]. At present, the research directions of TE materials are mainly divided into inorganic TE and organic TE (OTE) materials [5,6]. Compared with traditional inorganic TE materials, OTE materials has good mechanical flexibility and easy to process, giving them high potential applicability for wearable and large-area applications [7,8,9]. The thermal conversion efficiency of TE materials mainly depends on the size of the dimensionless figure of merit (*ZT*), *ZT = S^2^σT/κ*, where *S* is the Seebeck coefficient (thermopower), *σ* is the electrical conductivity, *T* is the absolute temperature, and *κ* is the thermal conductivity. OTE materials usually have low *κ* values (0.1–0.5 W m^−1^ K^−1^); therefore, the study of OTE materials is mainly focused on improving the value of the power factor (*S^2^σ*) [7].

Proper chemical doping for conjugated polymers (CPs) is an effective way to improve charge injection [10,11], such as doped CPs that can be used as conductive interlayers for organic light-emitting diodes (OLEDs) [12] and solar cells [13]. Similarly, chemical doping in an OTE material (i.e., by introducing a molecule that oxidizes or reduces copolymers to generate a free charge carrier) is also important [14]. During the doping process, the charge carrier transfers between dopants and polymers, and the concentration or mobility of the internal carriers will influence the Seebeck coefficient and electrical conductivity of CPs [15]. Recently, there are several reports of investigating the chemical doping process to improve the TE properties of CPs [16,17,18,19] For example, Jang et al. reported the F4TCNQ-doped 4*H*-cyclopenta[2,1-*b*:3,4-*b′*]dithiophene-based polymer (PCDTFBT) can achieve a high power factor up to 31.5 μW m^−1^ K^−2^ [20]. Yee et al. synthesized poly(3-alkylselenophenen) (P3RSe) to achieve a high power factor of 13 μW m^−1^ K^−2^ at higher dopant concentrations (FeCl_3_ ≈ 5 × 10^−3^ M) [19].

Although polymer doping has made great achievements, further developments in understanding the relationship between polymer structures and properties (e.g., carrier mobility, TE performance, and power conversion efficiency) are highly desirable to help us screen out valuable materials [20,21,22,23]. Moreover, a conjugated backbone is the basic unit that determines carrier mobility in CPs, by adjusting the intrinsic energy level and charge transfer efficiency between the CPs and dopants [24]. In addition, according to the continuous development of organic thermoelectric materials in recent years, enhanced thermoelectric performance has been obtained, but the reported results are mainly focused on the traditional conjugated polymers such as poly(3,4-ethylenedioxythiophene) (PEDOT), polypyrrole (PPy) and polyaniline (PANI). Ouyang, et al. recently reported the highest PF of PEDOT-PSS system which reached 155 mW/m^−1^ K^2^ [25]. The limitations of raw materials limit the development to a certain extent, therefore the screening of new potential thermoelectric materials are necessary for the discovery of high performance organic thermoelectric materials. 

Herein, we selected the indacenodithiophene (IDT) building block as the OTE material since the IDT skeleton has two thiophene rings attached with a central benzene ring that provide strong intermolecular interactions with ordered packing [26,27,28], which is beneficial for improving the electrical conductivity of OTE materials. IDT-based polymers have been widely used in organic field effect transistor (OFET) and solar cell applications [29,30,31]; however, although research on its TE properties has also been reported [32,33], the TE performance is still very low, and the detailed structure–property relationships are not comprehensive. In addition, we have recently reported IDT-based composite thermoelectric materials and found that the composites showed good performance with power factor of 161.34 mW/m^−1^ K^2^ [34]. 

**Figure 1:**
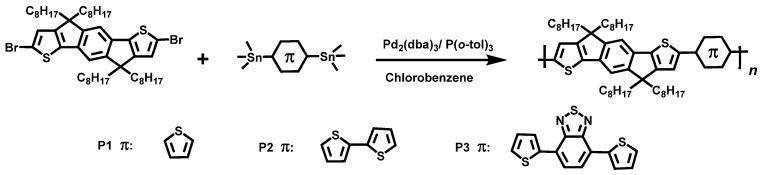
Chemical structures of the **IDT** unit and its copolymers.

In this work, we synthesized three IDT-based alternating copolymers **P1**, **P2**, and **P3** with different backbone structures to investigate the relationship between the structure-property and the chemical doping mechanism (Figure 1). Through a series of characterizations, we found that **P3** with D-A structure exhibits a two orders of magnitude higher carrier mobility (3.54 × 10^−3^ cm^2^ V^−1^ s^−1^) than that of **P1** and **P2**, and the higher carrier mobility makes **P3** to exhibit a higher electrical conductivity. The maximum power factor of **P3** reached 4.91 μW m^−1^ K^−2^ after p-type doping with FeCl_3_ at room temperature, which was much higher than the TE properties of **P1** and **P2**. These results indicate that the manipulation of CPs backbone structure is a promising strategy to develop high performance TE materials.

## 2. Experimental Section

### 2.1. Materials

2,7-Dibromo-4,4,9,9-tetraoctyl-4,9-dihydro-*s*-indaceno[1,2-*b*:5,6-*b’*]dithiophene, 2,5-bis(trimethylstannyl)thiophene, 2,2’-bis(trimethylstannyl)-5,5’-dithiophene and 4,7-bis(2-trimethylstannylthien-5-yl)-2,1,3-benzothiadiazole were purchased from Suna Tech Inc. (Suzhou, China). Tris(2-methylphenyl)phosphine (P(*o*-tol)_3_) and tris(dibenzylideneacetone) dipalladium(0) (Pd_2_(dba)_3_) were purchased from Greenchem Technlogy Co., Ltd. (Beijing, China). Anhydrous chlorobenzene (PhCl) and ferric chloride (FeCl_3_) were obtained from Sun Chemical Technology Co., Ltd. (Shanghai, China). Other chemical reagents—including deionized water, methanol, acetone, chlorobenzene, dichloromethane, and acetonitrile—were obtained from commercial sources, and all reagents were used without further treatment unless otherwise noted.

### 2.2. Synthesis of Polymers

#### 2.2.1. General Synthetic Procedure

A mixture of 2,7-dibromo-4,4,9,9-tetraoctyl-4,9-dihydro-*s*-indaceno[1,2-*b*:5,6-*b’*]dithiophene (0.200 g, 0.229 mmol), bis(trimethylstannyl) compound (0.229 mmol), Pd_2_(dba)_3_ (0.011 g, 0.011 mmol) and P(*o*-tol)_3_ (0.018 g, 0.057 mmol) in anhydrous chlorobenzene (5 mL) with a nitrogen flow was sealed and stirred for 72 h at 110 °C. After the mixture was cooled to room temperature, the polymer was precipitated by the addition of excess methanol. The precipitate was sequentially washed with methanol, acetone, and deionized water. After dried under vacuum, the polymer was obtained.

#### 2.2.2. 2-Methyl-7-(5-methylthiophen-2-yl)-4,4,9,9-tetraoctyl-4,9-dihydro-s-indaceno[1,2-b:5,6-b’]dithiophene (**P1**)

Brown solid (0.16 g, 66%). ^1^H NMR (600 MHz, CDCl_3_): *δ*/ppm 0.79–0.86 (t, 12H, CH_3_), 1.03–1.29 (m, 48H, CH_2_), 1.96 (d, 8H, CH_2_), 7.06–7.26 (m, 6H, aromatic). Analysis calculated for C_52_H_74_S_3_: C, 78.53; H, 9.38; S, 12.09. Found: C, 76.91; H, 10.19; S, 11.75.

#### 2.2.3. 2-Methyl-7-(5’-methyl-[2,2’-dithiophen]-5-yl)-4,4,9,9-tetraoctyl-4,9-dihydro-s-indaceno[1,2-b:5,6-b’]dithiophene (**P2**)

Brown solid (0.20 g, 76%). ^1^H NMR of **P2** (600 MHz, CDCl_3_): *δ*/ppm 0.81–0.84 (t, 12H, CH_3_), 0.85–1.33 (m, 48H, CH_2_), 1.95 (d, 8H, CH_2_), 6.99–7.20 (m, 6H, thiophene), 7.21–7.26 (s, 2H, aromatic). Analysis calculated for C_56_H_76_S_4_: C, 76.65; H, 8.73; S, 14.61. Found: C, 76.42; H, 9.65; S, 14.26.

#### 2.2.4. 4-(5-(7-methyl-4,4,9,9-tetraoctyl-4,9-dihydro-s-indaceno[1,2-b:5,6-b’]dithiophen-2-yl)thiophen-2-yl)-7-(5-methylthiophen-2-yl)benzo[c][1,2,5]thiadiazole (**P3**)

Black solid (0.24 g, 84%). ^1^H NMR (600 MHz, CDCl_3_): *δ*/ppm 0.80–0.87 (t, 12H, CH_3_), 0.87–1.51 (m, 48H, CH_2_), 1.94 (d, 8H, CH_2_), 7.22 (s, 2H, aromatic), 7.27–7.47 (m, 4H, thiophene), 7.92 (s, 2H, thiophene), 8.08 (s, 2H, aromatic). Analysis calculated for C_62_H_78_N_2_S_5_: C, 73.61; N, 2.77; H, 7.77; S, 15.85. Found: C, 72.84; N, 2.53; H, 8.47; S, 15.54.

### 2.3. Preparation of Polymer Films

**P1**, **P2**, and **P3** were dissolved in a chlorobenzene at a concentration of 5 mg mL^−1^. The pristine polymer films were obtained by drop-casting the solution onto glass substrates (10 × 10 mm, washed sequentially with dichloromethane, ethanol, acetone, and isopropanol for 30 mins) under ambient conditions. Film thicknesses of the prepared samples are ranged from 6 μm to 8 μm.

### 2.4. Doping Experiment

FeCl_3_ was dissolved in acetonitrile at a concentration of 0.1 M. The pristine polymer films were immersed in the FeCl_3_ solution for different times (1, 5, 10, 15, 20, and 30 min) under ambient conditions. Finally, the residual FeCl_3_ on the surface of the films was rinsed off with methanol.

## 3. Results and Discussion

**P1**, **P2**, and **P3** were synthesized by Stille coupling as shown in Figure 1. The structures of the polymers were confirmed by ^1^H NMR (Figure S1). The molecular weights of three polymers were determined by gel permeation chromatography (GPC), and the number-average molecular weights (*M*_n_) of **P1**, **P2**, and **P3** were found to be 52.8, 53.7, and 58.5 kDa, respectively (Figure S2). The thermal stability of the polymers was measured by thermal gravimetric analysis (TGA), as shown in Figure S3a, the decomposition temperature (*T*_d_, the 5% weight loss) of all polymers exceeded 380 ºC, indicating their good thermal stability. In particular, **P3** exhibits the highest thermal stability, most likely due to its rigid skeleton (Figure S3b and Table S1).

### 3.1. Optical and Electrochemical Characteristics

**Figure 2:**
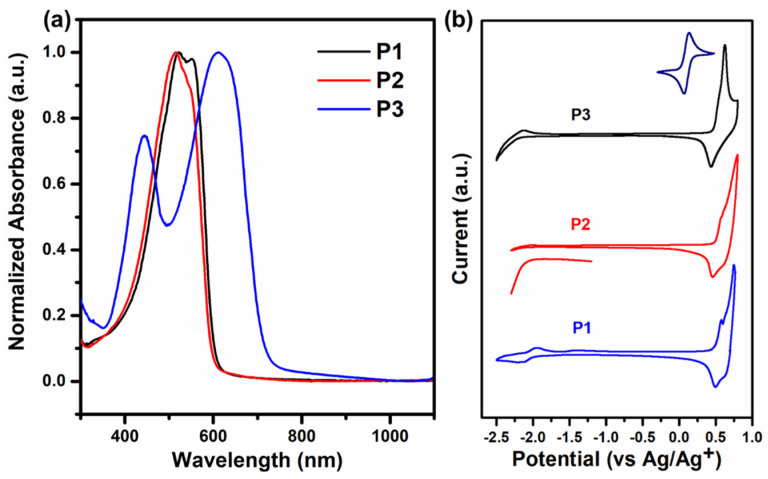
(**a**) UV–vis–NIR absorption spectra of non-doped **P1**–**P3** in film states. (**b**) Cyclic voltammograms of **P1**–**P3** in drop-casted films (the reduction peak of **P2** is amplified separately).

UV–vis–NIR absorption spectra of the three polymers in the non-doped thin-film state are shown in Figure 2a. Both **P1** and **P2** possessed similar spectral profiles with absorption maxima at 542 nm (**P1**) and 511 nm (**P2**), while **P3** exhibited two absorption peaks, the peak at 444 nm corresponds to the π–π* transition and the other peak at 625 nm should be attributed to the intramolecular charge transfer (ICT) from the electron-rich IDT segment to the electron-deficient benzothiadiazole unit [35,36]. The optical band gap (*E*_g_^opt^) values of the three polymers were calculated to be 2.02 eV (**P1**), 2.00 eV (**P2**), and 1.70 eV (**P3**), respectively. Cyclic voltammetry (CV) (Figure 2b) was performed to determine the highest occupied molecular orbital (HOMO) (*E*_HOMO_) and the lowest unoccupied molecular orbital (LUMO) (*E*_LUMO_) of the polymers according to the above-mentioned formula. The *E*_HOMO_/*E*_LUMO_ values of **P1**, **P2**, and **P3** were calculated to be −5.77/−3.21 eV, −5.78/−3.28 eV, and −5.77/−3.49 eV, respectively. It is clear to see that all the three polymers exhibit relatively deep HOMO energy levels. The *E*_g_^ec^ values of **P1**, **P2**, and **P3** were calculated to be 2.56, 2.50, and 2.28 eV, respectively (Table 1), which are in consistent with the trend of their optical bandgaps. The difference between the band gaps obtained by the two test methods is in accordance with the trend reported in the previous literature [37]. The relatively narrower bandgap of **P3** should be attributed to the charge transfer, providing the polymer to have a long effective conjugation length with more delocalized π-electrons [26].

**Figure 3:**
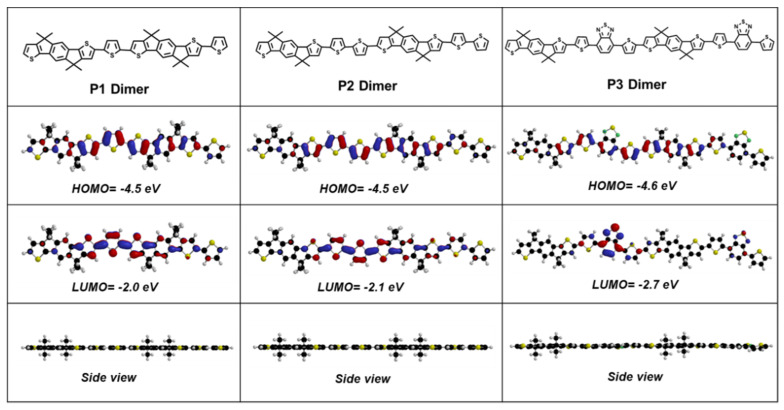
Molecular geometries and theoretical calculations for HOMO and LUMO energy levels of the model dimers of three polymers by density functional theory (DFT) calculations at the B3LYP/6-31G (d, p) level (The alkyl chain is replaced by the methyl group for clarity).

To further understand the electrochemical properties of the polymers and the distribution of delocalized electrons, we performed density functional theory (DFT) calculations on model dimers (Figure 3). The HOMO energy of the three polymers has similar delocalization electron distribution on the backbone, which means that the introduction of acceptor into the backbone of **P3** will not affect the delocalization electron distribution of HOMO energy. In contrast, the LUMO levels of **P1** and **P2** are delocalized on the entire polymer backbone, while the LUMO level of **P3** is mainly focused on the benzothiadiazole unit with electron deficient feature. The calculated LUMO levels for the three polymers were −2.00, −2.10, and −2.70 eV, respectively, we can see that **P3** showed a significantly deeper LUMO level compared to that of other two polymers, suggesting that the introduction of the D-A structure can deepen the LUMO energy to reduce the band gap (Table 1). These results are in good agreement with the experimental values from the CV measurements (i.e., *E*_g_^ec^). The side geometries of the polymers revealed the highly planar backbone features, which provides more evidence for the planar structure of three polymers that can facilitate the transfer of intermolecular charge carriers [24].

To investigate the mobilities of three polymers, we fabricated simple OFET devices without any optimizations (Figures S4 and S5), and the field effect mobility of the non-doped polymers was determined to be 3.48 × 10^−5^ (**P1**), 13.35 × 10^−5^ (**P2**), and 3.54 × 10^−3^ cm^2^ V^−1^ s^−1^ (**P3**). Although the mobility of **P3** is lower compared to the reported values in the previous reports due to the immature OFET devices [26], **P3** exhibited the highest carrier mobility among the three polymers investigated in this work, which is mainly due to its flat aromatic structure that can facilitate the intermolecular packing, thus enhancing the charge carrier transporting. Generally, CPs with higher carrier mobility is beneficial to larger electrical conductivity and higher is beneficial to larger Seebeck coefficients [39].

**Figure 4:**
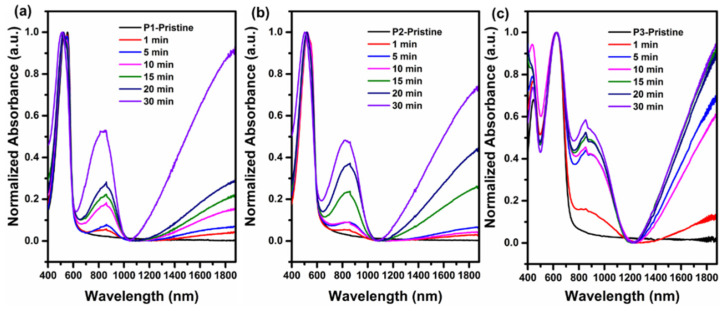
Time dependence of normalized UV–vis–NIR absorption spectra of polymer films doped with FeCl_3_: **P1** (**a**), **P2** (**b**), and **P3** (**c**).

UV–vis–NIR spectroscopy was also performed to investigate the extent of doping of the three polymers. As shown in Figure 4, after doping, new absorption peaks appeared at 872 (**P1**), 846 (**P2**), and 882 nm (**P3**) respectively with a broad peak at a wavelength over 1100 nm were observed, which should be generated from the formation of polarons [40,41]. As the doping time increased, the concentration of polarons were simultaneously increased.

### 3.2. Thin-Film Microstructure

**Figure 5:**
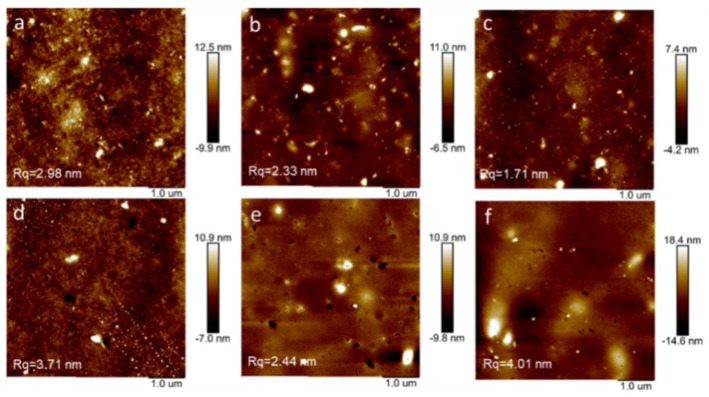
AFM images of **P1** (**a**), **P2** (**b**), and **P3** (**c**) pristine films and **P1** (**d**), **P2** (**e**), and **P3** (**f**) films doped with FeCl_3_ (0.1 M) for 15 min.

The introduction of dopants to polymer films usually causes microscopic changes in surface morphology, resulting in different localizations of the charge carriers in the polymers films [42]. Through GI-XRD and POM (Figure S6) analyses, we found that all three polymers are paracrystalline (see Supporting Information for details). As shown in Figure 5, the root mean square (RMS) values of the polymer films were obtained as follows: **P1** (2.98 nm), **P2** (2.33 nm), and **P3** (1.71 nm). **P3** exhibited a relatively smoother morphology compared to that of **P1** and **P2**. After doping (immersed in 0.1 M FeCl_3_/acetonitrile for 15 mins), all the three polymer films showed increased RMS values (**P1** (3.71 nm), **P2** (2.44 nm), and **P3** (4.01 nm)). It can be found that the surface roughness of **P1** and **P2** films only slightly increases after doping, compared to the roughness of **P3** films, which indicates that the stability of the morphology of **P1** and **P2** films during doping is higher than that of **P3** films. From the SEM (Figure S7), we can intuitively see that the surface of the polymer film after doping is still smooth. In the EDS (Figure S7), we also see that the residual iron element on the surface is evenly distributed without forming any aggregation, which also indicates that the FeCl_3_ doped the polymer uniformly.

### 3.3. Thermoelectric Performance

**Figure 6:**
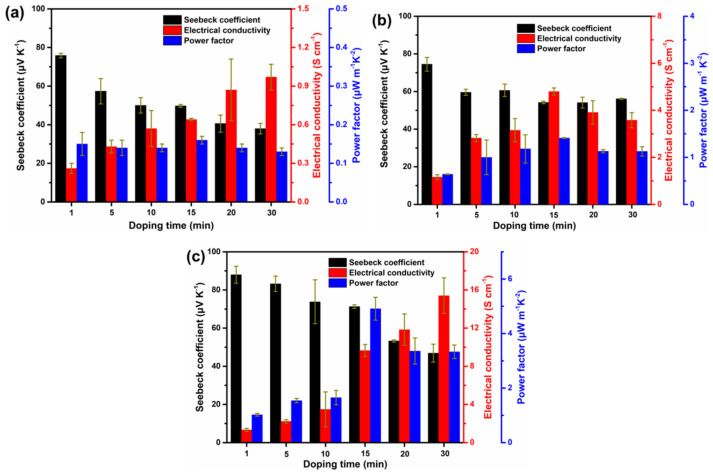
Thermoelectric performance for **P1** (**a**), **P2** (**b**), and **P3** (**c**) films with different doping times (immersed in the 0.1 M FeCl_3_/acetonitrile) at room temperature.

To compare the different TE properties, the pristine polymer films were immersed in 0.1 M FeCl_3_/acetonitrile for different doping times. We performed the measurements for multiple times under the same condition to ensure the reproducibility of the data. As shown in Figure 6, the Seebeck coefficient (*S*) and electrical conductivity (*σ*) showed opposite trends due to the enhanced concentration of polarons. After doping the polymers for 15 mins, **P1**, **P2**, and **P3** showed *σ* values of 0.64, 4.80, and 9.65 S cm^−1^, respectively. In the meantime, **P3** exhibited the largest value of Seebeck coefficient (*S*) (71.26 μV K^−1^) compared to those of **P1** (49.73 μV K^−1^) and **P2** (54.19 μV K^−1^). Therefore, **P3** showed the largest PF value of 4.91 μW m^−1^ K^−2^. In addition, we compared the recently reported thermoelectric properties of organic matter as shown in Table S2. Obviously, it should be noted that our material still has a certain gap compared with the composite TE material, but it shows higher TE performance compared with the performance of pristine polymer TE material after doping. Indicating that IDT-based conjugated polymers are promising materials for TE applications.

### 3.4. Photoelectron Spectroscopy

**Figure 7:**
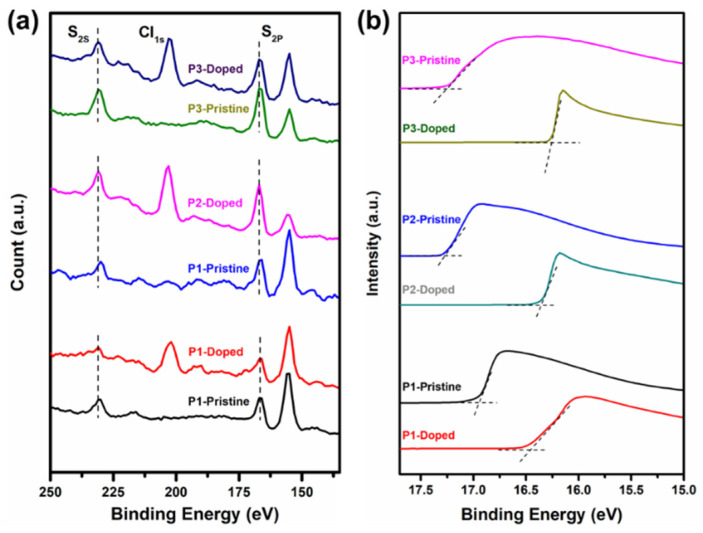
(**a**) XPS spectra S 2_S_ and S 2_P_ curves of pristine polymer films and films doped with FeCl_3_ (0.1 M) for 15 min; (**b**) UPS spectra (He I α radiation, photon energy = 21.22 eV) showing the secondary electron cut-off region for polymers in pristine and films doped with FeCl_3_ (0.1 M) for 15 min.

We investigated the electronic structure and doping mechanism of polymers through X-ray photoelectron spectroscopy (XPS) and ultraviolet photoelectron spectroscopy (UPS) (Figure 7). Compared to the results from the non-doped polymer, a new C l 1s peak derived from the dopant FeCl_3_ was observed. More importantly, the peaks of S 2s for **P1** and **P2** and S 2s and N 1s for **P3** (Figure S8) shifted toward a larger binding energy caused by the electron transfer occurs between the polymers and dopant [43]. This result proves that chemical doping of the polymer by FeCl_3_ is mainly achieved by oxidation of the bonding atoms in the polymer. In the UPS spectra (Figure S9), we found all the three polymers exhibited two different characteristic peaks. The peak in the range of 17.53–14.65 eV belongs to inelastic elastic electron scattering, and the peak in the range of 12.43–5.38 eV corresponds to the σ peak of the system [44]. Figure 7b shows the UPS secondary electron cut-off region of the polymer films in pristine and doped conditions. According to the formula (work function *ø* = 21.2 − *E*_cutoff_ eV), the work functions of **P1**, **P2**, and **P3** were calculated to be 4.24, 3.94, and 3.93 eV, respectively. For the secondary cutoff of the polymers, the voltage shifted to a smaller binding energy after doping, resulting in an increase in the work function (**P1**(4.72 eV), **P2** (4.86 eV), and **P3** (4.93 eV)). Combined with the energy level structure diagram shown in Figure S10, we conclude that the Fermi level moves towards the HOMO direction when the work function increases, indicating the generation of hole carriers, and also proves that FeCl_3_ is a p-type dopant for **P1**, **P2**, and **P3** [35,43]. The ionization energy (IE = *ø +* valence band (or HOMO) onset) [45] of **P1**, **P2**, and **P3** is 5.10, 5.01, and 5.18 eV, respectively (Figure S9d); the electron affinity of FeCl_3_ is 4.62 eV as documented in previous reports [11]. For p-dopable polymers, the doping efficiency should generally increase as the IE_polymer_ − (EA)_dopant_ difference increases, resulting in a larger thermodynamic driving force for polymer oxidation [46,47], therefore **P3** with the largest HOMO onset binding energy shows the best thermoelectric performance.

## 4. Conclusions

In this work, we synthesized three different IDT-based conjugated copolymers, **P1**, **P2**, and **P3,** to study how conjugated backbones affect the TE performances. We found that **P3** with a D-A alternating backbone exhibited a narrower band gap and higher carrier mobility than those of the other two polymers. Under the same doping conditions, **P3** showed the highest doping efficiency, which facilitates the charge transfer between the polymer and the dopant. As a result, **P3** exhibited a maximum PF of 4.91 μW m^−1^ K^−2^ at room temperature. These results prove that the IDT-based copolymers show great prospective applications as OTE materials, and a suitable backbone structural design is important for improving the TE properties.

## Figures and Tables

**Table 1:** Optical and electrochemical properties of the copolymers.

Polymer	*E*_HOMO_ (eV)	*E*_LUMO_ (eV)	*E*_g_^ec^ (eV)	*E*_g_^opt^ (eV)	*λ*_onset_ (nm)	*E*_g_^DFT^ (eV)	Carrier Mobility (10^−5^ cm^2^ v^−1^ s^−1^)
**P1**	−5.77	−3.21	2.56	2.02	613	2.50	3.48
**P2**	−5.78	−3.28	2.50	2.00	617	2.40	13.35
**P3**	−5.77	−3.49	2.28	1.70	730	2.10	354

*E*_g_^ec^: Electrochemical band gap. *E*_g_^opt^: Optical band gap.*λ*_onset_: Onset wavelength of the maximum absorption wavelength. *E*_HOMO_ = −(*E*_ox_ – *E*_1/2_^(Fc/Fc+)^) eV+ (−5.39 eV), *E*_LUMO_ = − (*E*_red_ – *E*_1/2_^(Fc/Fc+)^) eV + (−5.39 eV), *E*_g_^ec^ = *E*_LUMO_ – *E*_HOMO_, (Assuming that the formal potential of ferrocene is at 0.40 V versus SCE and that 0.24 V vs. NHE corresponds to 0.0 V vs. SCE, then the calibration value for ferrocene is −5.39 eV) [38].

## Supplementary Materials

The following are available online at https://www.mdpi.com/2073-4360/12/7/1463/s1.
