# Supplementary material for: Structure and Doping Optimization of IDT-Based Copolymers for Thermoelectrics
Source: Polymers (Basel). 2020 Jun 30;12(7):1463. doi: 10.3390/polym12071463 (PMC7407661; doi:10.3390/polym12071463)
Supplement: Supplementary file 1 [file polymers-12-01463-s001.pdf]

Supporting Information

Structure and doping optimization of IDT–based copolymers for thermoelectrics

Tongchao Liu [a, b‡], Dexun Xie [*, a], Jinjia Xu [c], and Chengjun Pan [b]

a) School of Chemistry, Sun Yat-sen University, Guangzhou, 510275, China

b) Shenzhen Key Laboratory of Polymer Science and Technology, College of Materials Science and Engineering, Shenzhen University, Shenzhen 518060, China.

c) Weldon School of Biomedical Engineering, Birck Nanotechnology Center, Center for Implantable Devices, Purdue University, West Lafayette, IN 47907, USA.

‡ These authors contributed equally to the work.

Emails: xiedx9@mail.sysu.edu.cn

Instruments and Measurements

^1H NMR spectra of the polymers were acquired on an AVANCE III 600 MHz NMR spectrometer (Bruker) at room temperature in $CDCl_3$. Chemical shifts are expressed in part per million (ppm, δ scale) and are referenced to tetramethylsilane (TMS). Thermal gravimetric analysis (TGA) was performed on a TGA-55 instrument (TA Instruments) from room temperature to 700 °C under a nitrogen flow of 20 mL min^{-1} with a heating rate of 10 °C min^{-1}. The molecular weights and polydispersity index (PDI) of the polymers were determined by gel permeation chromatography (GPC) (Waters) using THF as the eluent, and polystyrene was used as a standard. Differential scanning calorimetry (DSC) was performed on a DSC7020 instrument (Hitachi), in which three polymers were measured in a temperature range from 0 ºC to 300 ºC at a heating rate of 10 ºC min^{-1}. Ultraviolet–visible–near-infrared (UV–vis-NIR) absorption spectra were acquired using a Lambda 950 spectrophotometer (PerkinElmer). The morphology of the polymer films was observed by a SU-70 scanning electron microscope (SEM) (Hitachi), and the thickness of the films was obtained on a Surfcorder ET 4000M (Kosaka Laboratory, Japan). Tapping-mode atomic force microscopy (AFM) images of the polymer films were obtained using Dimension ICON (Bruker) to observe the roughness of the film surface. Cyclic voltammetry (CV) was performed on a CHI 660E electrochemical workstation (CH Instruments). Platinum plate, Ag/Ag^+ and a platinum wire were used as the working, reference and counter electrodes, respectively, in 0.1 M tetrabutylammonium hexafluorophosphate (Bu4NPF6)/acetonitrile under a nitrogen atmosphere at a scan rate of 50 mV s^{-1}. The reference electrode was calibrated with ferrocene/ferrocenium (Fc/Fc^+). Polarized optical microscopy (POM) was performed on an ECLIPSE LV100N POL microscope (Nikon). Grazing incidence X-ray diffraction (GI-XRD) was measured on a SmartLab X-ray diffractometer (Rigaku,

Japan) with a copper target (λ = 1.54 Å), and the incident range was 2-40 °. X-ray photoelectron spectroscopy (XPS) data were obtained by a field-emission auger spectrometer (Thermo Fisher ESCALAB 250X). Ultraviolet photoelectron spectroscopy (UPS) measurements were tested on an ESCALAB 250XI photoelectron spectrometer (Thermo Fischer Scientific) under a vacuum of approximately 2 × 10^{-8} Torr with a He I α gas discharge lamp (21.22 eV) and monochromatic Al Kα as the excitation sources. The electrical conductivity (σ) and Seebeck coefficient (S) of the polymer films were collected using an MRS-3 thin-film thermoelectric test system (Wuhan Joule Yacht Science & Technology, China).

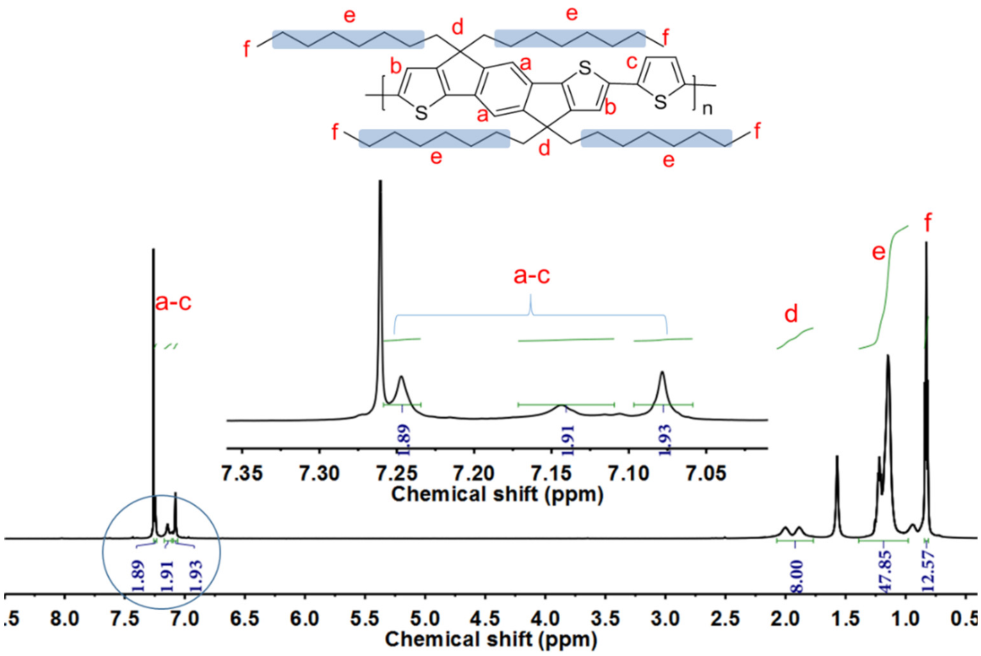

(a)

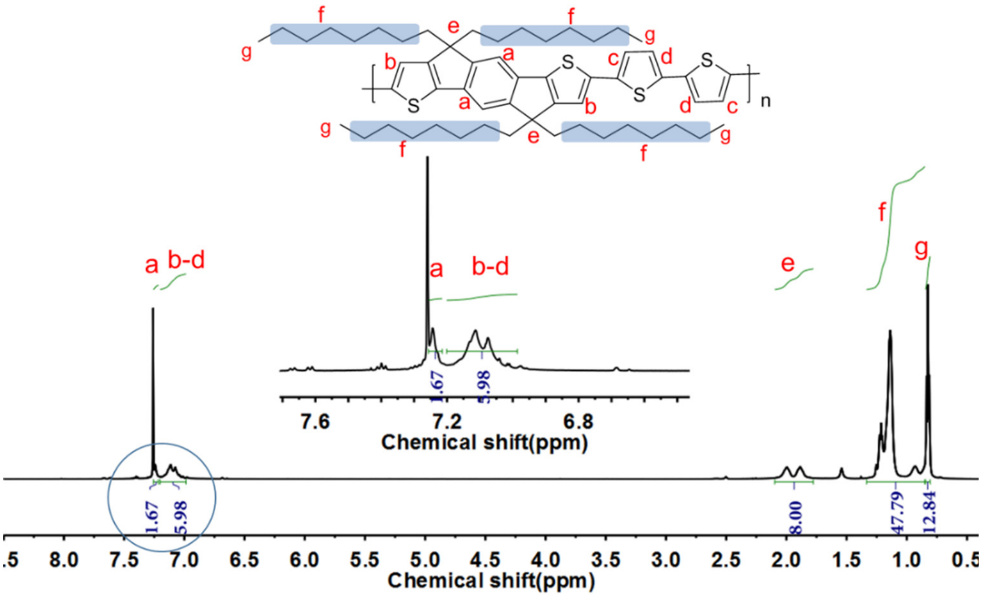

(b)

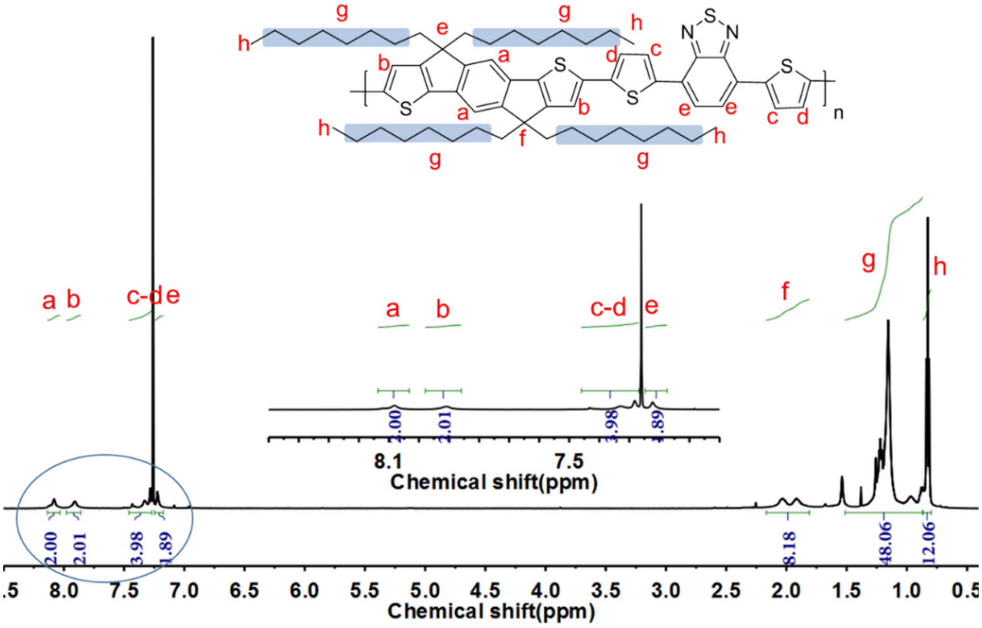

(c)

Figure S1. ^{1}H NMR (600 MHz, $CDCl_3$) spectra of (a) **P1**, (b) **P2** and (c) **P3**.

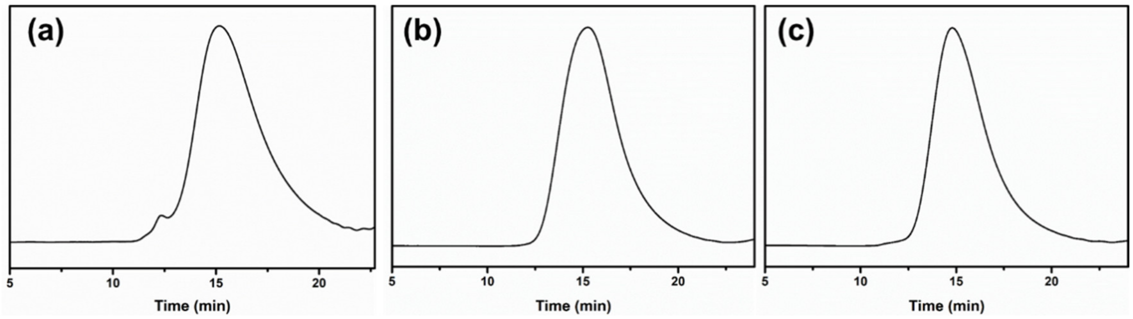

Figure S2. GPC curves of (a) **P1**, (b) **P2** and (c) **P3**.

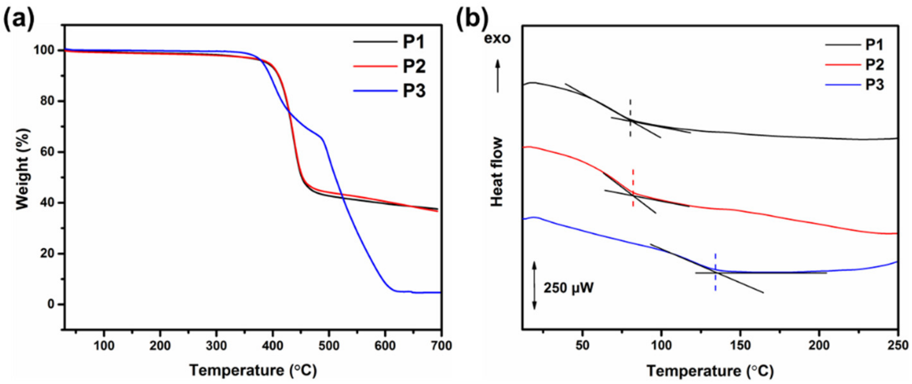

Figure S3. TGA curves of polymers (a) and DSC curves of polymers (b).

Table S1. Molecular weights and thermal properties of polymers.

Polymer	M_n (kDa)	M_w (kDa)	PDI	DP_n[a]	T_d[b] (°C)	T_g[c] (°C)
**P1**	52.83	112.52	2.13	67	389	74
**P2**	53.71	121.92	2.27	61	392	76
**P3**	58.54	132.70	2.27	58	401	133

a Degree of polymerization determined on the basis of the number of repeating monomer units. b Polymer decomposition temperature. c Polymer glass transition temperature.

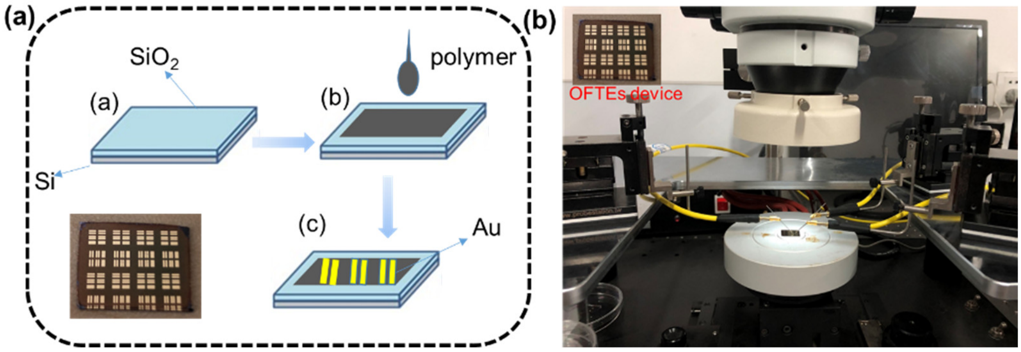

Figure S4. (a) The fabrication process of the OFET device, (b) its test process

P1, **P2** and **P3** were dissolved in chlorobenzene at a concentration of 5 mg mL^{-1}. After the polymer was evenly dispersed in chlorobenzene, it was uniformly dropped on the surface of a silicon single-sided polished sheet (surface coated with a layer of 100 nm thick silicon oxide). The polymer coated silicon wafer is then placed in an organic optoelectronic device deposition system and uniformly plated with a thickness of about 21 nm of gold (Au) to obtain a simple organic field-effect transistors (OFETs) device. Finally, the device is placed on the probe station, silicon and silicon oxide are used as the substrate and dielectric, while the polymers are used as semiconductor layers. The Au electrodes are used as the source and the drain, then the carrier mobility of the polymer-based OFETs was tested in a Keithley 4200A-SCS parametric analyzer.[1,2] The typical transfer I–V curves of the best-performing drop-cast OTFTs as measured at room temperature are shown in Figure S5a–c, respectively. And the on/off ratio of the three OFETs are: 4.82 (P1), 8.96 (P2) and 55.23 (P3).

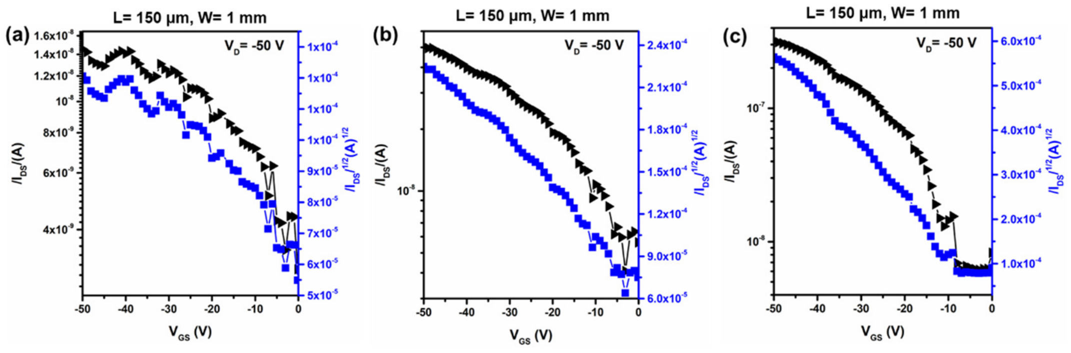

Figure S5. Transfer current–voltage (I–V) curves for the best-performing: (a) **P1**, (b) **P2**, (c) **P3**.

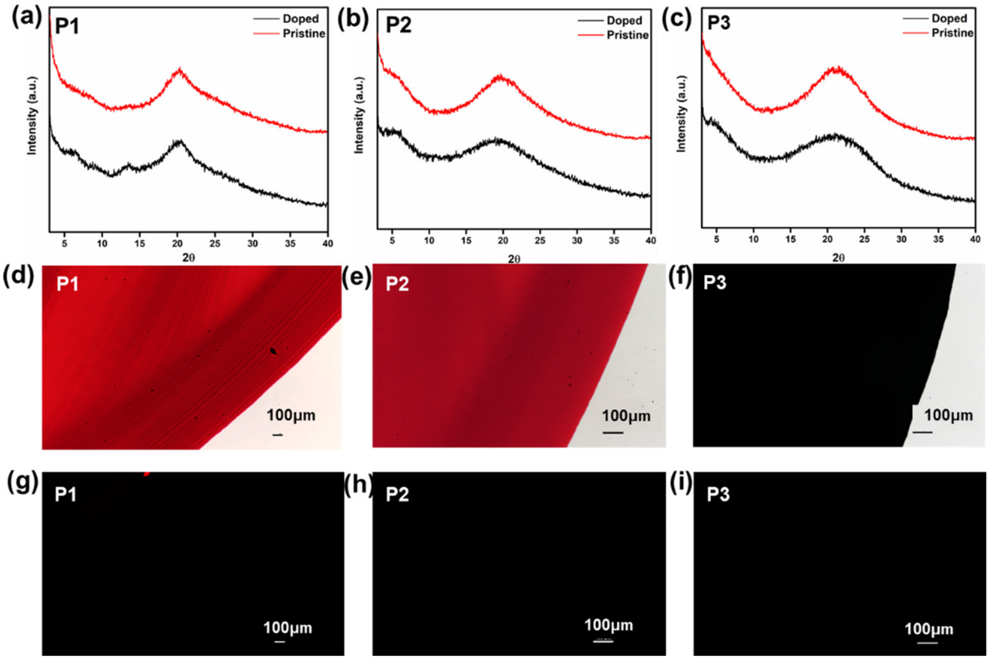

Figure S6. GI-XRD (a–c) curves of polymer films under pristine and doped with $FeCl_3$ (0.1 M) for 15 min; Optical microscopic (d–f) and polarized microscopic (g–i) images of polymer films at pristine conditions.

GI-XRD measurement is used to investigate the crystalline properties of three polymers in film states. As shown in **Figure S6**, no obvious peaks were found at region from 2 º to 15 º, indicating that no ordered packing formed in polymers,[3] which is consistent with the measurements observed by polarized optical microscopy. **P1**, **P2** and **P3** exhibited wide diffraction peaks at 2θ = 20.14 º, 20.24 º

and 21.17 º, respectively. According to the Bragg equation $2d\sin\theta = n\lambda$, the corresponding inter-layer d-spacings are calculated to be 4.40 Å, 4.38 Å and 4.19 Å, respectively.[4] This result suggested that **P3** exhibits denser molecular packing, owing to the introduction of the D-A structure and decrease the intramolecular stacking distance.[5] As previously reported, compact molecular structures can lead to higher carrier mobility of the polymer as evidenced by OFET testing.[6,7]

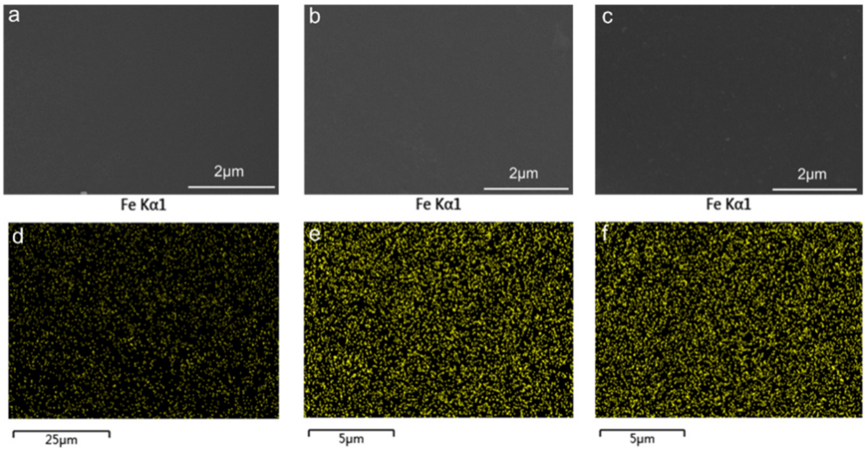

Figure S7. SEM images of **P1** (a), **P2** (b) and **P3** (c) films doped with $FeCl_3$ (immersed in the 0.1 M $FeCl_3$/acetonitrile for 15 min); EDS images of polymer doped with $FeCl_3$ (0.1 M) for 15 min: **P1** (d), **P2** (e) and **P3**.

Table S2. A comparison of thermoelectric performance of organic thermoelectric.

Sample	Dopant	*PF* (μW m^{-1} K^{-2})	Ref.
**P3**	$FeCl_3$	4.91	This work
**IDT-polymer/SWCNT**	-	161.34	[8]
**PEDOT:PSS**	$Ti_3C_2T_x$	155	[9]
**BBTa26**	F4TCNQ	11.80	[10]

**P3HT**	F4TCNQ	8	[11]
**PIDTT-EDOT**	$FeCl_3$	0.87	[12]
**PpPD**	HCl	0.56	[13]
**PEDOT**	S-PHE	0.53	[14]
**IDT-TT**	$FeCl_3$	0.47	[15]

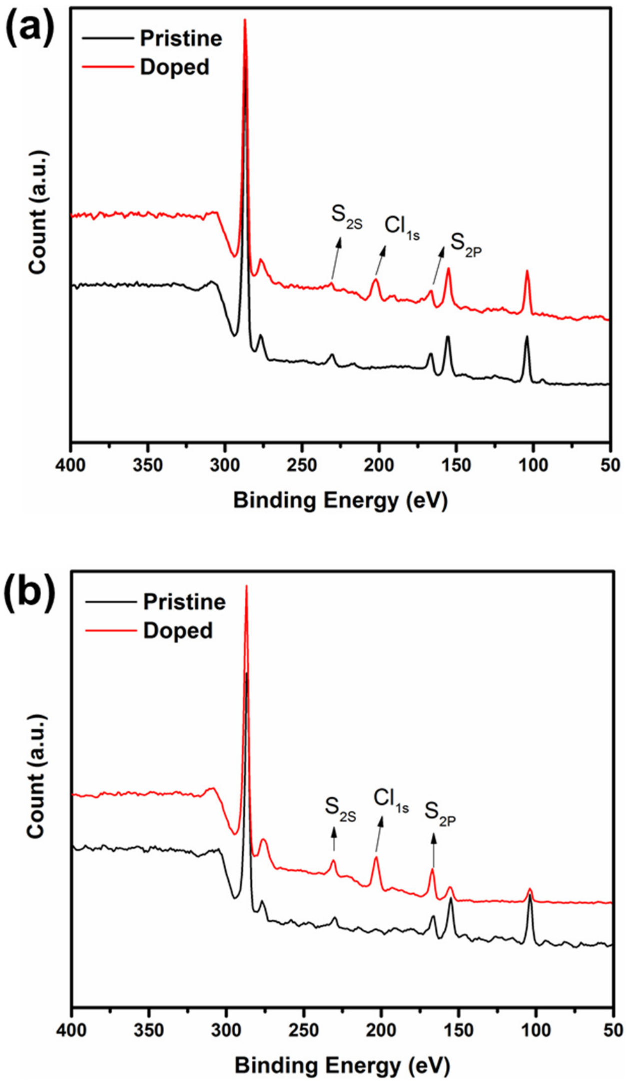

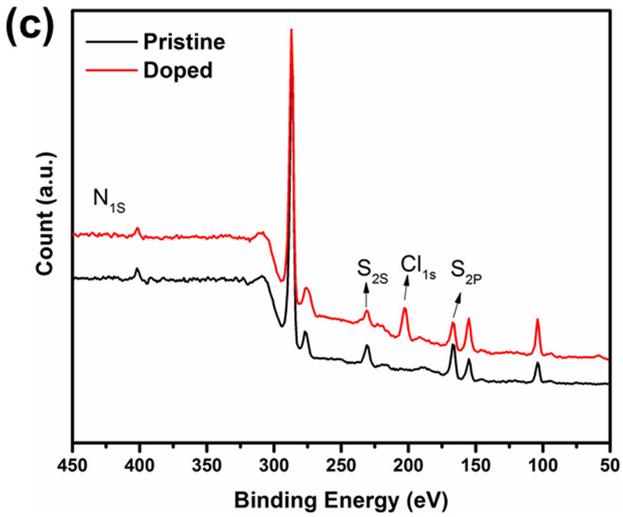

Figure S8. XPS curves of pristine polymer films and doped with $FeCl_3$ (0.1 M) for 15 min: (a) **P1**, (b) **P2**, (c) **P3**.

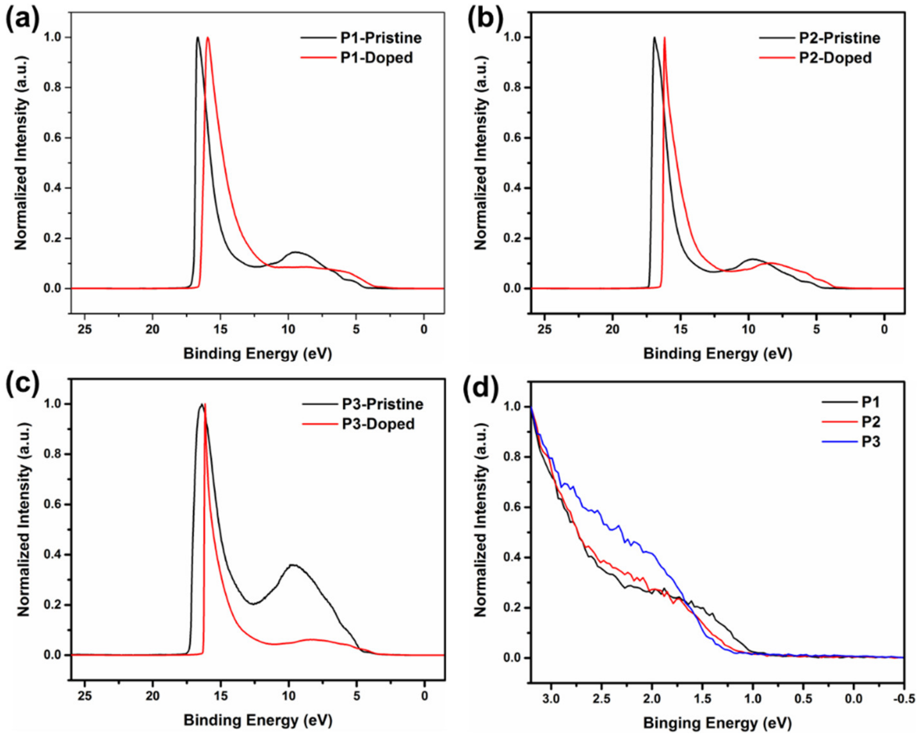

Figure S9. UPS valence band spectra (He I α radiation) of pristine polymer film and polymer film doped with $FeCl_3$ (0.1 M) for 15 min: (a) **P1**, (b) **P2**, and (c) **P3**. (d) The valance band regions of polymers in pristine conditions.

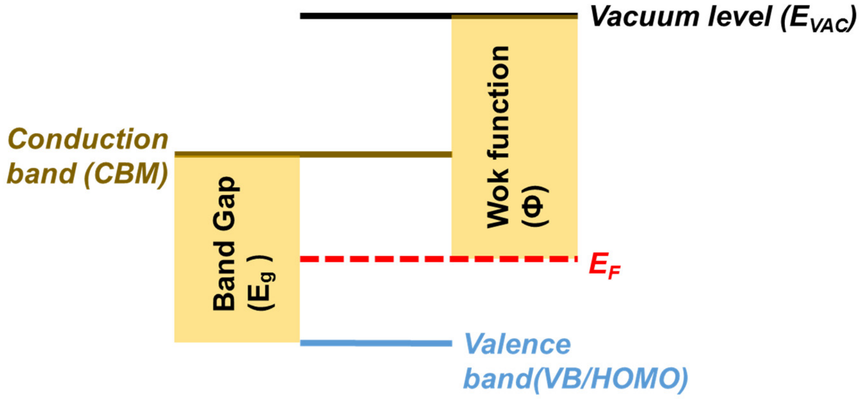

Figure S10. Simulated image of conduction band, valence band and fermi level structure.

Reference

(1) Ma, H.; Yip, H. L.; Huang, F.; Jen, A. Interface Engineering for Organic Electronics. *Adv. Funct. Mater*. **2010**, *20*, 1371–1388.

(2) Yin, Z.; Yin, M.; Liu, Z.; Zhang, Y.; Zhang, A.; Zheng, Q. Solution-Processed Bilayer Dielectrics for Flexible Low-Voltage Organic Field-Effect Transistors in Pressure-Sensing Applications. *Adv. Sci*. **2018**, *5*, 1701041.

(3) Hou, J. Huo, L.; He, C.; Yang, C.; Li, Y. Synthesis and Absorption Spectra of Poly(3-(phenylenevinyl)thiophene)s with Conjugated Side Chains. *Macromolecules* **2006**, *39*, 594–603.

(4) Zhang, S.; Ye, L.; Zhao, W.; Liu, D.; Yao, H.; Hou, J. Side Chain Selection for Designing Highly Efficient Photovoltaic Polymers with 2D-Conjugated Structure. *Macromolecules* **2014**, *47*, 4653–4659.

(5) Sun, Y.; Chien, S.; Yip, H.; Zhang, Y.; Chen, K.; Zeigler, D.; Chen, F.; Lin, B.; Jen, A.

Hig-mobility low-bandgap conjugated copolymers based on indacenodithiophene and thiadiazolo units for thin film transistor and photovoltaic applications. *J. Mater. Chem*. **2011**, *21*, 13247–13255.

(6) Kim, D.; Park, Y.; Jang, Y.; et al. Enhancement of field-effect mobility due to surface-mediated molecular ordering in regioregular polythiophene thin film transistors. *Adv. Funct. Mater*. **2005**, *15*, 77–82.

(7) Kline, R.; McGehee, M.; Kadnikova, E.; Liu, J.; Frechet, J.; Toney, M. Dependence of regioregular poly(3-hexylthiophene) film morphology and field-effect mobility on molecular weight. *Macromolecules* **2005**, *38*, 3312–3319.

(8) Chen, Z.; Liu, T.; Pan, C.; Tan, G. Enhanced Thermoelectric Performance of Indacenodithiophene Benzothiadiazole Copolymer Containing Polar Side Chains and Single Wall Carbon Nanotubes Composites. *Polymers*. **2020**, *12*, 848.

(9) Guan, X.; Feng, W.; Wang, X.; Venkatesh, R.; Ouyang, J.; Significant Enhancement in the Seebeck Coefficient and Power Factor of p-Type Poly(3,4-ethylenedioxythiophene):Poly(styrenesulfonate) through the Incorporation of n-Type MXene. *ACS. Appl. Mater. Inter*. **2020**, *12*, 13013–13020.

(10) Tam, T.; Ng, C.; Lim, S.; Yildirim, E.; Ko, J.; Leong, W.; Yang, S.; Xu, J. Proquinoidal-Conjugated Polymer as an Effective Strategy for the Enhancement of Electrical Conductivity and Thermoelectric Properties. *Chem. Mater*. **2019**, *31*, 8543–8550.

(11) Zuo, G.; Andersson, O.; Abdalla, H.; Kemerink, M. High thermoelectric power factor from multilayer solution-processed organic films. *Appl. Phys. Lett*. **2018**, *112*, 083303.

(12) Wei, C.; Wang, L.; Pan, C.; Chen, Z.; Zhao, H.; Wang, L.; Effect of backbone structure

on the thermoelectric performance of indacenodithiophene-based conjugated polymers. *React. Funct. Polym*. **2019,** *142*, 1–6.

(13) Chen, Y.; Kou, Q.; Cai, K.; Yin, J.; Shen, S. The influence of hydrochloric acid doping on the microstructure and thermoelectric properties of poly(p-phenylenediamine) nanoparticles. *Synthetic Met*. **2017**, *227*, 71–77.

(14) Imae, I.; Koumoto, T.; Harima, Y. Thermoelectric properties of polythiophenes partially substituted by ethylenedioxy groups. *Polymer* **2018**, *144*, 43–50.

(15) Wu, S.; Wu, X.; Xing, W.; Sun, Y.; Zou, Y.; Xu, W.; Zhu, D. Backbone Structure Effect on the Thermoelectric Properties of IDT-Based p-Type Conjugated Polymers. *Macromol. Rapid. Commun*. **2019**, e1900322.
